# Controlled deep breathing technique intervention effects on resting EEG, subjective psychological distress, and executive function performance in young adults

**DOI:** 10.3389/fpubh.2026.1952693

**Published:** 2026-09-29

**Authors:** Ohav Ivgi, Oded Meiron

**Affiliations:** Brain and Memory Laboratory, Faculty of Education, Bar-Ilan University, Ramat Gan, Israel

**Keywords:** alpha oscillations, cognitive control, controlled breathing, emotion regulation, qEEG

## Abstract

Controlled breathing techniques have been widely investigated for their role in emotional regulation; however, their effects on resting-state neural oscillatory activity and psychological distress remain insufficiently characterized. The present study examined whether a Controlled Deep Breathing Technique (CDBT) intervention modulates resting-state electroencephalographic (EEG) activity and subjective psychological distress in healthy adults. Forty healthy adults (ages 18 to 40 years) were randomly assigned to an experimental (CDBT) or control (natural breathing) intervention group for 20 min. Resting-state quantitative EEG (qEEG) was recorded before and after the intervention under eyes-open and eyes-closed conditions. Behavioral measures included assessments of subjective psychological distress and executive functioning. qEEG analyses during resting-state focused on alpha/beta power ratios at frontal and parietal electrodes. Results showed a significant increase in alpha/beta ratios in the experimental group compared with the control group, particularly during the eyes-closed condition and at parietal electrodes. The experimental group also showed significant reductions in psychological distress scores and improvements in executive functioning measures, whereas no significant changes were observed in the control group. These findings suggest that CDBT may modulate frontoparietal oscillatory dynamics associated with relaxed mental states and improved executive functioning, providing preliminary evidence that CDBT may influence emotional regulation and cognitive control in healthy adults.

## 1 Introduction

Controlled breathing techniques have gained increasing attention in recent years due to their potential effects on emotional regulation, physiological arousal, and cognitive functioning. Growing evidence indicates that slow, paced breathing can influence autonomic nervous system (ANS) activity by reducing sympathetic activation and increasing parasympathetic dominance, thereby promoting relaxation and stress reduction. These physiological mechanisms have been associated with improvements in emotional well-being and top-down attentional processes (1).

Despite the growing interest in breathing-based interventions, fewer studies have examined their direct effects on neural activity using electrophysiological methods. Resting-state electroencephalography (EEG) provides a valuable tool for examining spontaneous quantitative changes in neural oscillations associated with different cognitive and emotional states. Among the dominant neural oscillatory activity observed during resting periods, alpha oscillations (8–12 Hz) are commonly associated with relaxed wakefulness, attentional control, and emotional regulation. Increased resting alpha activity has been linked to reduced stress levels and improved inhibitory control during increased cognitive demands (2, 3). However, examining alpha power as a correlate of change in behavior alone may not fully capture the balance between neural excitation and inhibition across frontoparietal networks. For this reason, recent research has highlighted the importance of examining the ratio between alpha and beta activity. Beta oscillations (13–30 Hz) are typically associated with active cognitive processing, heightened alertness, and increased mental effort (4). The alpha/beta power ratio has therefore been proposed as a sensitive neurophysiological marker reflecting shifts between relaxed and more active, cognitively engaged neural states (5). In healthy adults, higher alpha/beta ratios have been associated with improved emotional regulation, reduced anxiety, and more efficient recovery (e.g., coping behaviors) from stress.

Controlled breathing techniques may influence neural oscillatory activity through their effects on the autonomic nervous system (ANS). Slow-paced, deep-breathing interventions have been shown to increase parasympathetic activity and support emotional regulation through autonomic mechanisms (1, 6, 7). These effects may influence neural oscillatory activity and cognitive control processes, potentially through changes in alpha activity (2, 3). For instance, Jerath et al. (6) reported that slow breathing practices enhance vagal tone and are associated with increased resting alpha activity, primarily observed over frontal (Fz, F3, F4) and parietal (Pz, P3, P4) electrodes. These changes may reflect reduced physiological arousal or enhanced relaxation, potentially involving autonomic mechanisms. Furthermore, neuroimaging studies using fMRI and PET have demonstrated that such breathing practices modulate activity in key neocortical and limbic cortex areas, including the prefrontal cortex and the insula, respectively, which are involved in emotional regulation and ANS top-down control (1).

Previous research suggests that breathing-based practices can increase alpha power and influence physiological stress-related responses (8) (e.g., heart rate variability and electrodermal activity), indicating a shift toward more relaxed neural states during wakefulness following controlled deep-breathing training (6, 9). An additional factor relevant to resting-state quantitative EEG (qEEG) research in humans concerns differences in the mean power of oscillations at specific frequency bands (e.g., alpha frequency band) between eyes-open and eyes-closed conditions. Classic qEEG studies have consistently demonstrated that eyes-closed relative to eyes-open states are associated with increased alpha power and reduced beta power, particularly over posterior regions (2, 10). These consistently observed differences in alpha power between eyes-closed and eyes-open conditions reflect systematic shifts in neural network recruitment, indicating differences in sensory processing and mental arousal between externally oriented and internally oriented states (11). Beyond neural activity or reactivity to external stimuli, breathing interventions may also influence behavioral outcomes related to emotional distress perception and executive functioning in humans.

The present study examined the effects of a Controlled Deep Breathing Technique (CDBT) on resting-state qEEG features (e.g., mean alpha/beta power ratio), emotional distress states, and executive functioning in healthy adults. Participants were randomly assigned to either an experimental group practicing CDBT or a control group engaging in natural breathing. Resting-state EEG activity was recorded before and after the intervention period under both eyes-open and eyes-closed conditions, focusing on alpha/beta power ratios and alpha power at frontal and parietal regions. Therefore, we hypothesized that participants in the experimental group would show an increase in alpha power and a higher alpha/beta power ratio following the CDBT intervention compared with the control group intervention, potentially reflecting neural states associated with reduced mental arousal and relaxation. In addition, it was expected that the experimental group would demonstrate reduced self-reported distress symptoms/state-related distress and improved cognitive control performance compared with the control group from pre- to post-intervention.

## 2 Methods

### 2.1 Participants

Participants included in the study were right-handed, native Hebrew-speaking healthy adults aged 18–40 years (*n* = 40). They were randomly assigned to either an experimental group (*n* = 20) or a control group (*n* = 20). Inclusion criteria required participants to be in good physical and mental health, with no history of neurological, psychiatric, sensory, or developmental disorders. Screening was conducted using a comprehensive medical history questionnaire, including items assessing neurological and psychiatric history, current medication use, previous psychological or psychiatric treatment, history of head injury or loss of consciousness, and overall physical health status. All participants provided written informed consent prior to participation. The study was approved by the Human Subjects Institutional Review Board of Bar-Ilan University.

An *a priori* power analysis was conducted using G*Power (version 3.1.9.7) to determine the required sample size for detecting a between-group effect in a mixed experimental design (8). The justification for the effect size was based on prior empirical EEG studies examining the effects of breathing-based and mindfulness interventions on alpha-band activity. Bing-Canar et al. (9) investigated the effects of a mindful breathing exercise on EEG activity during a cognitive task in a sample of approximately 62 participants and found a significant increase in alpha-band activity compared with a control condition, with a large effect size (ηp^2^ = 0.18). In addition, Perciavalle et al. (4) examined the effects of deep breathing exercises within a mixed experimental design in a sample of approximately 38 participants and reported a significant effect of the intervention on psychophysiological measures related to relaxation and attentional regulation, with an effect size of ηp^2^ = 0.13. Power analysis was conducted using an alpha level of α = 0.05 and power (1 − β) = 0.80. Following power analysis, a sample size of 34 participants was expected to detect a medium effect size (Cohen’s *f* = 0.25); therefore, we recruited 40 participants to account for possible dropouts or missing data following EEG analysis.

### 2.2 Study design and procedure

The study employed a randomized single-blind controlled design to examine the effects of the Controlled Deep Breathing Technique (CDBT) on resting-state brain activity, emotional distress, and executive functioning. Participants were randomly assigned to either CDBT or control group using a simple randomization procedure. After providing informed consent and filling out demographic and medical questionnaires, participants underwent a baseline assessment phase that included a resting-state EEG recording and behavioral assessments. Resting-state EEG recordings were obtained under alternating eyes-open (EO) and eyes-closed (EC) conditions. In the eyes-open conditions, participants visually fixated on a central cross (“+”) displayed on the screen. Each condition lasted one minute and alternated for a total of six minutes of recording (three EO-EC cycles). Following the baseline assessment phase, participants completed a 20-min intervention session (CDBT versus Control). Immediately after the intervention, participants underwent a second resting-state EEG recording identical to the baseline phase. Participants completed the same behavioral assessments (as in the baseline phase) following the post-intervention EEG recording session.

### 2.3 Breathing intervention (CDBT versus control)

During the single-session intervention, participants were seated comfortably in a quiet testing room for approximately 20 min. Participants in the experimental group practiced the Controlled Deep Breathing Technique (CDBT), which consisted of a paced breathing cycle of 6 s inhalation, 4 s breath holding, and 4 s exhalation, repeated continuously throughout the session (1, 6). Standardized verbal instructions were provided by the experimenter to ensure adherence to the breathing rhythm. Participants in the control group were instructed to sit quietly, remain awake, and breathe naturally without receiving any explicit breathing instructions for a period of 20 min.

### 2.4 Depression Anxiety Stress Scales – 21 items (DASS-21)

Psychological distress was assessed using the Depression Anxiety Stress Scales – 21 items (DASS-21), a self-report questionnaire measuring three negative-affect dimensions of perceived psychological distress in humans such as depression, anxiety, and stress. Participants rated the extent to which each statement related to subjective-affective-state on a 4-point scale ranging from 0 (did not apply to me at all) to 3 (applied to me very much or most of the time). Total scores were computed, with higher scores indicating greater levels of distress. The DASS-21 has demonstrated strong internal consistency (α = 0.93) and good construct validity (10). Although the scale is typically designed to assess subjective emotional states over the preceding two weeks, prior intervention studies have shown that it can be sensitive to short-term, affective state-related changes in psychological distress following breathing- and relaxation-based interventions (e.g., (1)).

### 2.5 Trail Making Test (TMT)

Executive functioning was assessed using the Trail Making Test (TMT), a widely used neuropsychological measure of visual attention and cognitive flexibility (11). The test consists of two parts. Part A requires participants to connect numbered circles in ascending order (using a pencil-and-paper diagram) as quickly as possible without errors, whereas Part B requires alternating between numbers and letters in ascending order as quickly as possible without errors (e.g., 1-A-2-B). Primary outcome measures included completion time and accuracy (number of errors), with errors recorded for further analysis in Part B.

### 2.6 EEG acquisition and analysis

Resting-state EEG activity was recorded using a wireless 32-channel EEG system (EPOC Flex, Emotiv Inc., USA). EEG electrodes were positioned according to the international 10–20 system, nested within a reusable elastic EEG cap (EasyCap, Germany). The cap included 34 electrodes (electroplated Ag/AgCl electrodes with replaceable polyester felt pads soaked with saline and contacting the skin), including two reference channels (common-mode sensor [CMS] on the left and driven-right-leg [DRL] sensor on the right) positioned at the left and right mastoid locations, and 32 scalp EEG electrodes. Signal quality monitoring (contact and impedance values across all electrodes) was performed before and during EEG acquisition using EmotivPRO software (version 4.8.39.601), including real-time inspection of contact quality and impedance levels displayed through a visual interface on the computer screen. Impedance was maintained below 5 kΩ across all EEG recordings. EEG recording signal resolution was 14 bits (1 LSB = 0.51 μV; 16-bit ADC, with 2 bits of instrumental noise floor discarded). EEG acquisition employed a maximum slew rate of 32.64 μV/sample and a dynamic range of 4.12 μV. The acquisition settings included a high-pass filter at 2 Hz, a low-pass filter at 45 Hz (with a 50 Hz notch filter), a sampling rate of 1,024 Hz, a 5th-order digital sinc filter, and downsampling to 128 Hz. These steps were applied to reduce environmental noise and enhance signal quality. EEG data were collected during alternating eyes-open (EO) and eyes-closed (EC) resting intervals over a total duration of six minutes (three EO–EC cycles). During the EO condition, participants visually fixated on a central cross displayed on a computer screen for 60 s, whereas during the EC condition, they were instructed to close their eyes and remain relaxed and awake for 60 s. The Emotiv EPOC Flex EEG system (Emotiv Inc., USA) is a wireless EEG system used for recording resting-state EEG and ERP data in humans across different research settings. It has been shown to provide reliable recordings of resting-state oscillations and sensory ERP components and to produce data comparable to research-grade wired EEG systems commonly used in human EEG research (12).

Raw EEG data were preprocessed and analyzed using EmotivPRO Analyzer software (Emotiv Inc., USA). Standard preprocessing procedures included automatic artifact rejection for eye movements and muscle activity. Only artifact-free EEG epochs were retained for frequency-power analysis during EO and EC periods. Quantitative EEG (qEEG) analyses were conducted using Fast Fourier Transformation (FFT) to compute normalized mean power in the alpha (8–13 Hz), low beta (13–20 Hz), and high beta (21–30 Hz) frequency bands across all clean EEG epochs and all 32 channels. Mean normalized oscillatory power (μV^2^/Hz) for each target electrode and frequency band was averaged across one-second clean epochs during both eyes-open and eyes-closed resting conditions. Additional electrodes (Fp1, Fp2, P7, and P8) were included for internal electrode comparisons but were not included in the primary statistical analysis.

### 2.7 Statistical analysis

Resting-state qEEG and behavioral measures were collected at pre- and post-intervention time points. Dependent variables included DASS-21 total scores, Trail Making Test (TMT) performance, and alpha/beta power ratios (Alpha/BetaL and Alpha/BetaH) measured at frontal and parietal electrode sites. For qEEG measures, a mixed-design ANOVA was conducted with Time (pre, post), Eyes Condition (eyes open, eyes closed), Scalp Region (frontal, parietal), and Ratio Type (alpha/betaL, alpha/betaH) as within-subject factors, and Group (experimental, control) as a between-subjects factor. For assessing effects on behavioral measures (DASS-21 and TMT), separate 2 × 2 mixed-design ANOVAs were conducted with Time (pre to post) as a within-subject factor and Group (experimental, control) as a between-subjects factor.

Prior to statistical analyses, assumptions of normality and homogeneity of variance were assessed using the Shapiro–Wilk and Levene’s tests, respectively. Outliers were defined as values exceeding ±3 standard deviations within each group score distribution (13). All statistical tests were two-tailed with α = 0.05. Effect sizes are reported as partial eta squared (η^2^_p_) for ANOVA effects and Cohen’s d for pairwise comparisons. Based on this criterion, one participant was identified as an outlier and excluded from further analyses, resulting in a final sample of 39 participants.

## 3 Results

### 3.1 Participants

The final analyzed sample consisted of 39 participants, including 20 in the control group and 19 in the experimental group. A chi-square test indicated no significant difference in gender proportion between groups (χ^2^(1) = 0.03, *p* = 0.87). In the control group, 50% of participants were male and 50% were female, whereas in the experimental group 52.6% were male and 47.4% were female. The mean age in the control group was 25.15 years (SD = 3.47), compared with 25.05 years (SD = 3.67) in the experimental group. An independent-samples *t*-test confirmed that this difference was not statistically significant (*t*(37) = 0.09, *p* = 0.93). Similarly, no significant group differences were observed for years of education. Participants in the control group had a mean of 14.55 years of education (SD = 1.85), whereas participants in the experimental group had a mean of 14.42 years (SD = 1.84) (*t*(37) = 0.22, *p* = 0.83).

### 3.2 Resting-state alpha/beta power ratios

A mixed-design ANOVA was conducted to examine the effects of Time (pre, post), Eyes Condition (eyes-open, eyes-closed), Scalp Region (frontal, parietal), Ratio Type (alpha/betaL, alpha/betaH), and Group (experimental, control) on resting-state alpha/beta power ratios. The analysis revealed a significant main effect of Time (*F* (1, 37) = 21.73, *p* < 0.001, η^2^_p_ = 0.364), indicating an overall increase in alpha/beta ratios from pre- to post-intervention. Importantly, a significant Group × Time interaction was observed (*F* (1, 37) = 16.83, *p* < 0.001, η^2^_p_ = 0.307), indicating that the increase in alpha/beta power ratios was significantly greater in the experimental group compared to the control group across frontal and parietal electrodes of interest (EOI). A significant main effect of Eyes Condition (eyes-open vs. eyes-closed) was also found (*F* (1, 37) = 21.80, *p* < 0.001, η^2^_p_ = 0.364), with higher alpha/beta power ratios observed during the eyes-closed (EC) condition compared to the eyes-open (EO) condition (Figure 1).

**Figure 1:**
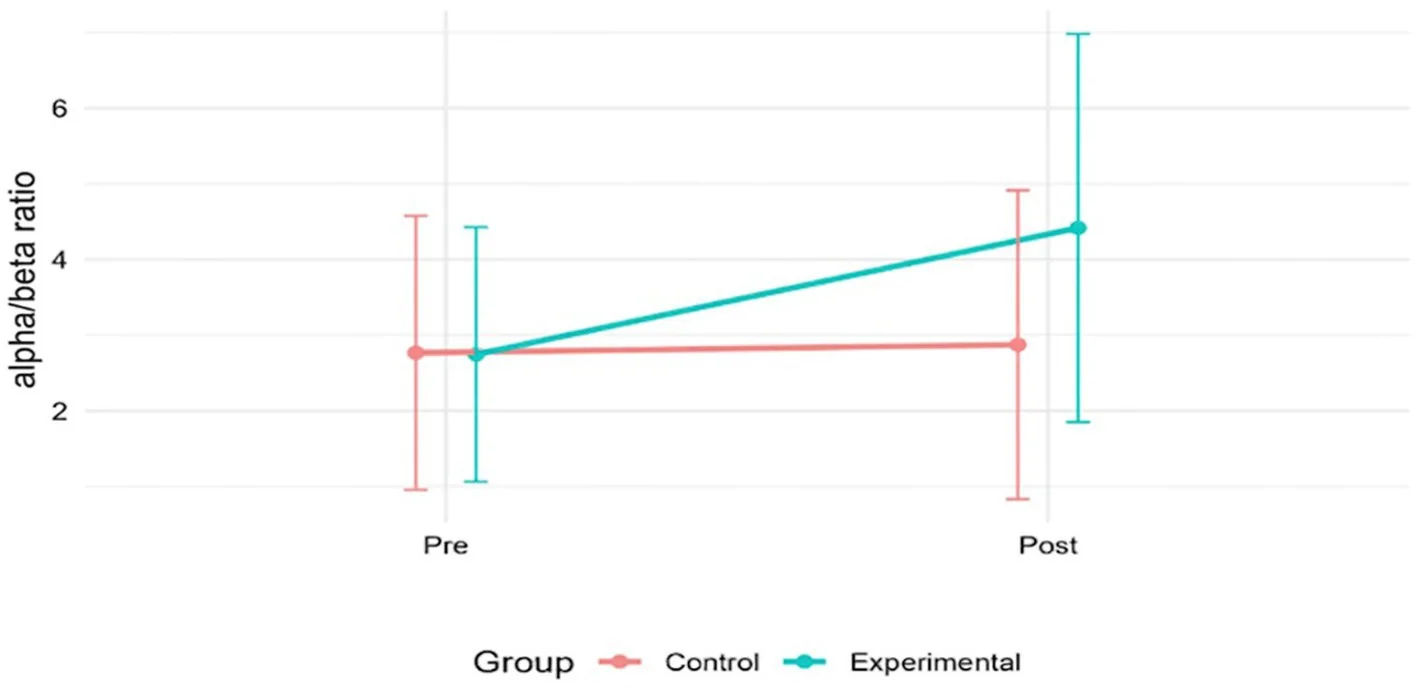
Mean alpha/beta power ratio as a function of group and time. The figure presents mean alpha/beta power ratios averaged across all electrodes of interest (EOI: Fz, F3, F4, Pz, P3, and P4) during pre- and post-intervention assessments in the experimental and control groups. Vertical bars represent ±1 standard deviation (SD).

Furthermore, a significant Group × Time × Scalp Region interaction was detected (*F*(1, 37) = 5.80, *p* = 0.021, η^2^_p_ = 0.132), reflecting greater change in alpha/beta ratios over parietal electrode sites compared to frontal regions (Figure 2). Follow-up pairwise comparisons across frontal and parietal electrodes of interest showed that alpha/beta power ratios significantly increased only within the experimental group, from pre-intervention (M = 2.74, SD = 0.29) to post-intervention (M = 4.42, SD = 0.36) (*t* (18) = 6.20, *p* < 0.001, Cohen’s d = 1.42). In contrast, the control group did not show a significant change over time, with ratios changing only minimally from pre-intervention (M = 2.77, SD = 0.29) to post-intervention (M = 2.87, SD = 0.36) (t (19) = −0.59, *p* = 0.56, Cohen’s d = 0.13) (Figure 2).

**Figure 2:**
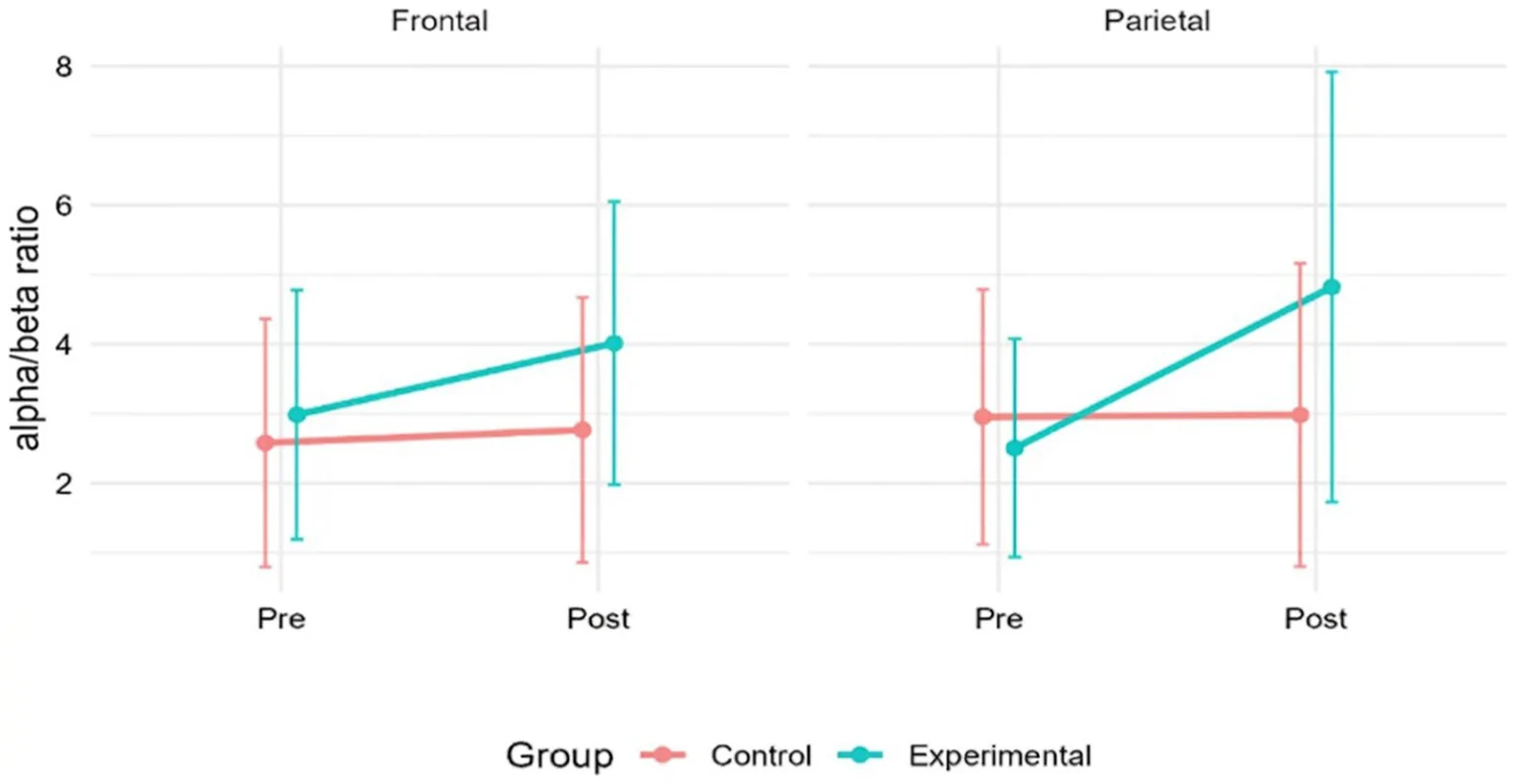
Group × time × region interaction for the combined alpha/beta power ratio across frontal and parietal electrodes. The figure presents changes in alpha/beta power ratios across frontal and parietal electrodes of interest over time in the experimental and control groups. Error bars represent ±1 standard deviation (SD).

Additional analyses were conducted to determine whether the observed increase in alpha/beta power ratios was driven by increased alpha-band power, decreased beta-band power, or both. Results indicated that the increase in alpha/beta ratios in the experimental group was primarily driven by increased alpha power, as reflected by a significant Group × Time interaction for alpha activity (*F*(1, 37) = 11.61, *p* = 0.002, η^2^_p_ = 0.234). Changes in beta activity were comparatively small, with low-beta power showing minimal nonsignificant changes across conditions and high-beta activity remained stable across time and groups (*p* > 0.05). These findings suggest that the increase in alpha/beta ratios primarily reflects enhanced alpha oscillatory activity rather than reductions in beta activity. Topographically, the increase in alpha activity appeared more pronounced over parietal electrodes (Pz, P3, P4) than over frontal sites, particularly during eyes-closed conditions.

In order to explicitly examining *alpha reactivity* to determine if CDBT effects are specifically associated with changes in visual cortical excitability directly related to neurophysiological response of eyes-closed conditions versus open-eyes conditions (e.g., EC-EO difference before and after intervention) under target parietal electrodes, we computed the difference in alpha power under target parietal electrodes (Pz, P3, P4) between closed to open eyes conditions from pre to post intervention across all participants. We employed a mixed-ANOVA to examine the effects of group and time on alpha reactivity (EC-EO alpha power difference) using the mean alpha-difference power values as the main dependent variable. A significant main effect of time was found showing that that mean alpha reactivity was higher following intervention (*F*(1, 38) = 12.59, *p* = 0.001, η^2^_p_ = 0.24). Importantly, a significant interaction emerged between time and group (*F* (1, 38) = 5.87, *p* = 0.02, η^2^_p_ = 0.13). The interaction indicated that the CDBT group showed a significant increase in alpha reactivity versus the control group, that showed no change in alpha reactivity over time (see Figure 3). *Post hoc* paired-sample t-tests within each group examining alpha reactivity from pre (M = 0.53, SD = 0.83) to post (M = 1.96, SD = 2.13) intervention confirmed this selective time effect only in the CDBT group (t (17) = 2.484, *p* = 0.024, Cohen’s d = 0.57). In contrast, the control condition showed no significant difference in alpha reactivity from pre ( M=−0.204,SD=2.313 ) to post ( M=0.800,SD=1.808 ), $t\;(19)=1.358,p=.190,{\text{Cohen}}^{'}s\;d=0.304\;\text{intervention}$ . Figure 3 illustrates this selective time-effect on alpha reactivity only in the experimental group versus the control group.

**Figure 3:**
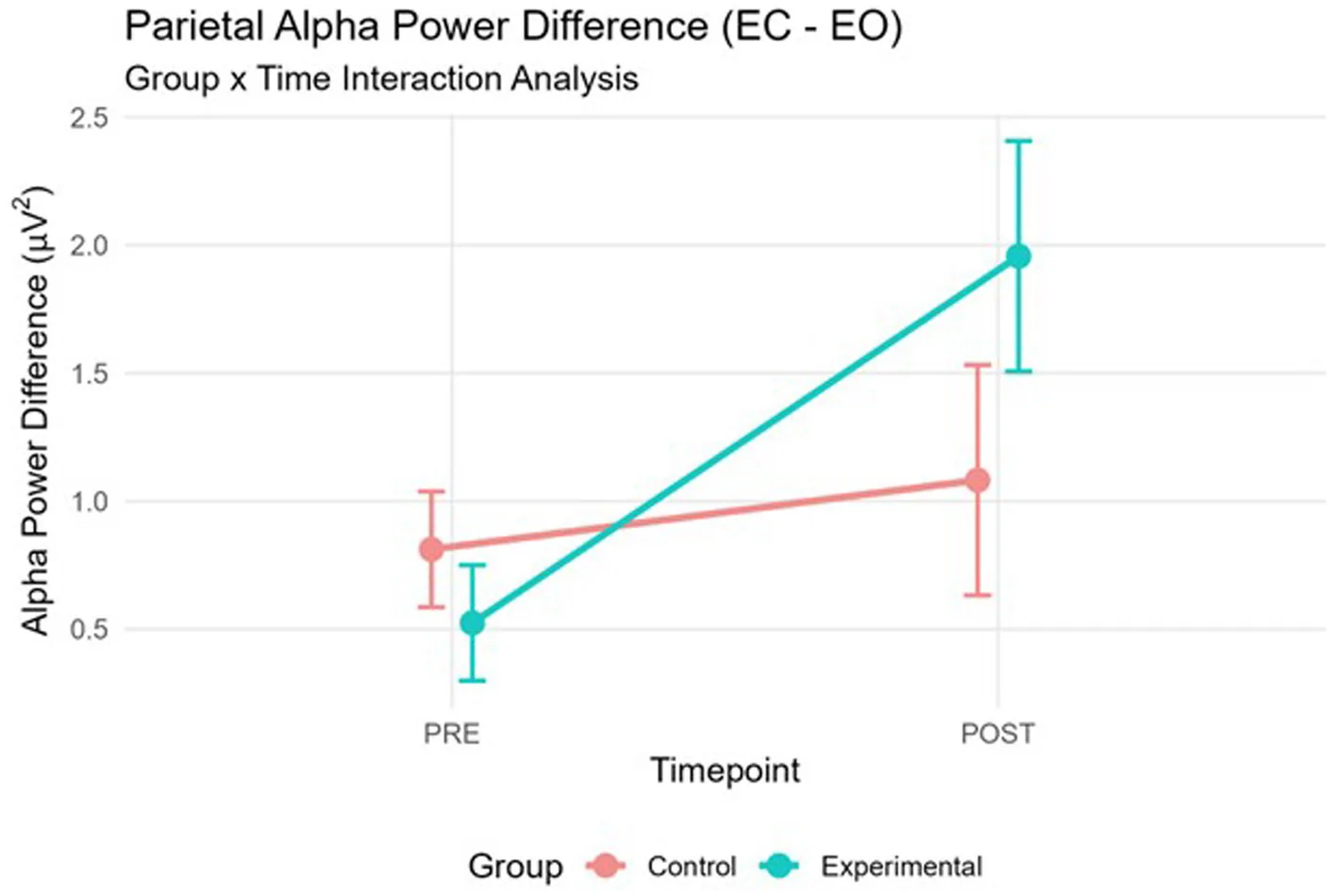
Group × Time interaction effect on alpha reactivity. The figure presents mean alpha reactivity, calculated as the difference in alpha power between the eyes-closed and eyes-open conditions (EC − EO), at target parietal electrodes (Pz, P3, and P4) before and after the intervention in the experimental and control groups. Error bars represent ±1 standard deviation (SD).

### 3.3 Psychological distress (DASS-21) scores

A mixed-design ANOVA examining DASS-21 total scores revealed a significant main effect of time (*F* (1, 37) = 12.25, *p* = 0.001, η^2^_p_ = 0.244), indicating an overall decrease in subjective psychological distress scores from pre- to post-intervention. Importantly, a significant Group × Time interaction was observed (*F* (1, 37) = 40.01, *p* < 0.001, η^2^_p_ = 0.513), indicating that the reduction in distress was significantly greater in the experimental group compared to the control group (Figure 4). *Post hoc* paired-sample t-tests confirmed a significant decrease in DASS-21 scores in the experimental group, with scores decreasing from pre-intervention (M = 29.70, SD = 7.59) to post-intervention (M = 23.00, SD = 8.53) (t (18) = −9.14, *p* < 0.001, Cohen’s d = 2.10). In contrast, no significant change was observed in the control group (t (19) = −0.59, *p* = 0.56, Cohen’s d = 0.13), with scores changing only minimally from pre (M = 29.80, SD = 5.94) to post-intervention (M = 29.40, SD = 6.49). These findings suggest that the breathing intervention was associated with reduced psychological distress, particularly in the experimental group.

**Figure 4:**
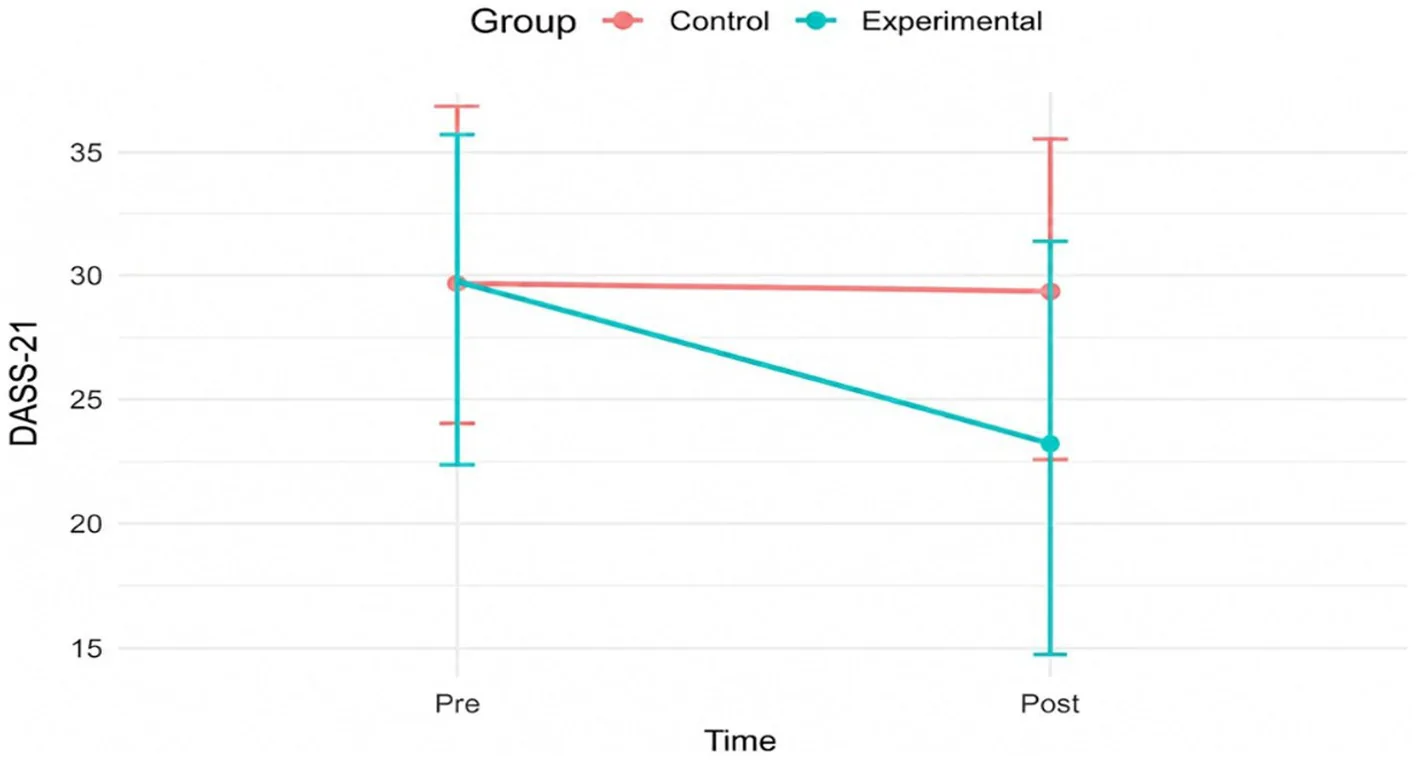
Group × Time interaction effect on DASS-21 total scores. The figure presents mean DASS-21 total scores before and after the intervention in the experimental and control groups. Error bars represent ±1 standard deviation (SD).

### 3.4 Cognitive performance (Trail Making Test–Part B)

A significant main effect of Group was also observed for TMT-B completion times (*F* (1, 37) = 10.42, *p* = 0.003, η^2^_p_ = 0.215), suggesting improved performance in the experimental group compared to the control group after intervention (Figure 5).

**Figure 5:**
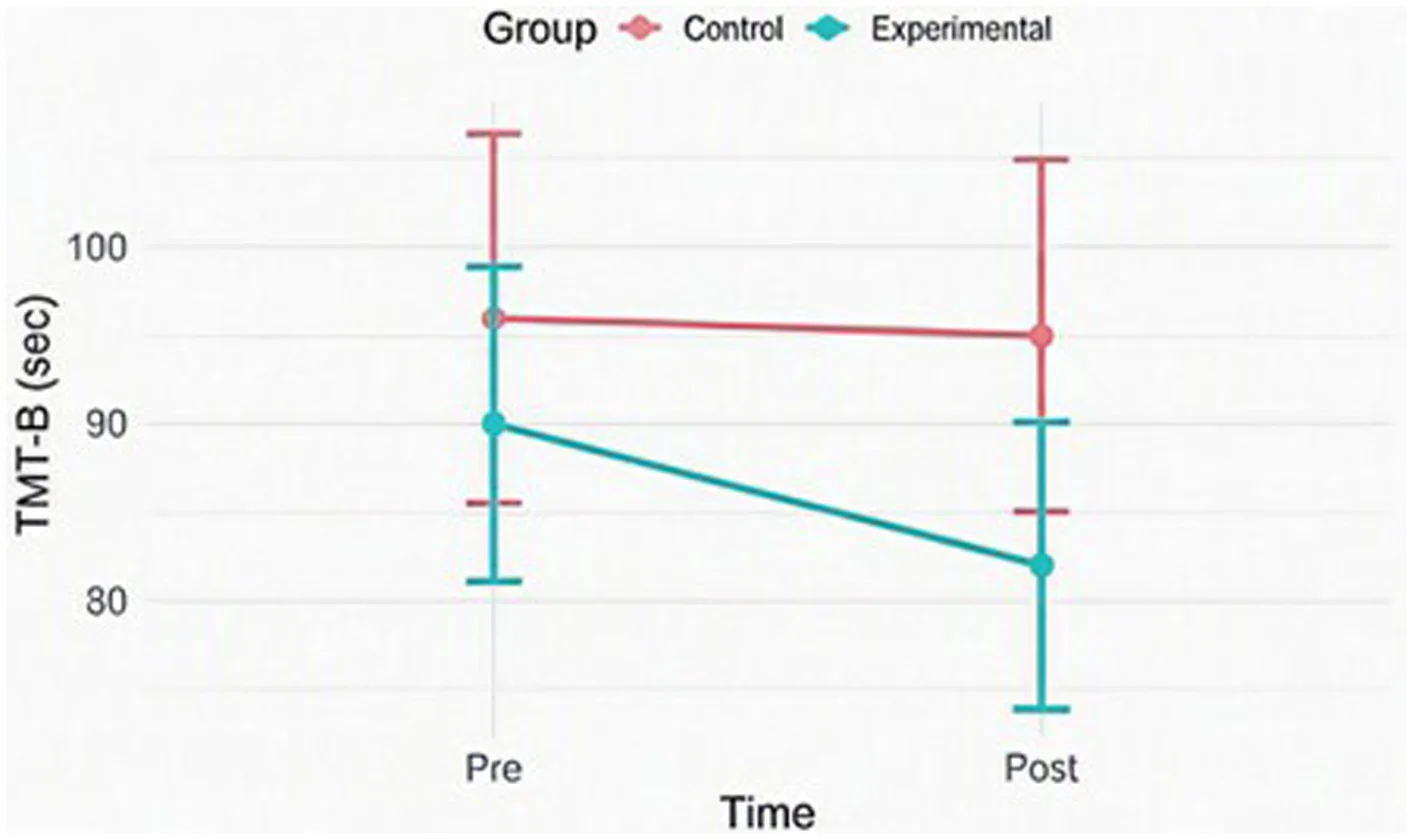
Group × Time interaction for Trail Making Test–Part B (TMT-B) completion times. The figure presents mean TMT-B completion times before and after the intervention in the experimental and control groups. Error bars represent ±1 standard deviation (SD).

Follow-up paired-sample *t*-tests showed that participants in the experimental group completed the task significantly faster following the intervention, with completion times decreasing from pre-intervention (M = 89.89, SD = 8.90) to post-intervention (M = 81.93, SD = 8.10) (t (18) = −13.59, *p* < 0.001, Cohen’s d = 3.12), reflecting improved cognitive flexibility and attentional visual–spatial control. The control group showed a statistically significant but small change over time, with a small effect size observed (t (19) = −2.24, *p* = 0.038, Cohen’s d = 0.50), which may reflect subtle practice effects.

## 4 Discussion

The present study examined the effects of a Controlled Deep Breathing Technique (CDBT) on resting-state qEEG parameters, psychological distress, and executive functioning in healthy adults. The current findings indicate that even a brief breathing intervention or brief CDBT induction may produce measurable changes in neural activity and cognitive functioning in healthy young adults. Consistent with the study hypotheses, participants who practiced controlled deep breathing demonstrated a significant increase in mean alpha/beta power ratio during specific resting-state intervals. This effect was particularly pronounced during the eyes-closed condition and was primarily evident over parietal electrodes of interest (EOI). In contrast, participants in the control group who engaged in natural breathing did not show significant changes in resting-state neural activity. Overall, these findings suggest that CDBT may contribute to changes in resting-state neural activity, executive functioning, and psychological distress compared with a natural-breathing control condition.

Importantly, our results indicated that a rise in alpha/beta power ratios was primarily driven by elevated alpha power rather than reduced beta power. Changes in alpha oscillations have been widely associated with relaxed wakefulness, internally directed attention, and efficient cognitive processing. Increased alpha activity has also been linked to reduced physiological arousal and improved emotional regulation (2, 3). The present findings therefore suggest that controlled deep breathing may influence resting neural states associated with relaxed mental states and attentional processes. However, further studies incorporating direct physiological measures are required to clarify the underlying mechanisms. In addition, our findings regarding CDBT effects on EEG and behavioral measures should be interpreted with caution, due to possible differences in uncontrolled variables such as sustained attentional engagement or differences in sensory-specific CBDT effects (e.g., verbal instructions), we cannot exclude attentional-allocation effects that were not controlled in the control group. Significant and more pronounced CDBT effects on alpha activity were observed particularly during the eyes-closed condition, consistent with earlier resting-state qEEG research demonstrating that alpha activity increases when external visual input is reduced (13, 14, 26). During eyes-closed conditions, internally oriented neural processes become more dominant. The parietal predominance of increased alpha activity was primarily evident in the experimental group, indicating larger post-intervention changes in alpha activity over parietal compared with frontal EOI, whereas no comparable effects or regional modulation were observed in the control group. Although changes in resting alpha activity are associated with changes in relaxed wakefulness states, event-related alpha oscillatory activity has been widely associated with multiple neurocognitive processes in human-EEG literature, including sensory inhibition, functional gating, internally directed attention, cortical excitability, and attentional allocation (15–17). Thus, our additional analysis related to changes over time in *alpha reactivity* (denoted by changes in posterior alpha power during close-eyes minus posterior alpha power during open-eyes conditions) indicated an increase in alpha reactivity only in the CDBT group following intervention. Accordingly, CDBT intervention resulted in a post-intervention increase in the amplitude and reactivity of the dominant posterior alpha oscillations. This finding supports and extends previous research showing posterior alpha is naturally dominant during resting eyes-closed conditions across healthy humans (25), particularly versus open-eyes conditions (related to visual cortical-pathway excitability), and that we can behaviorally modify these specific resting neurophysiological states in humans, as well as benefiting mood and executive function performance (22, 27).

Beyond resting neural activity, the breathing intervention also produced significant effects on psychological distress and executive functioning scores in the experimental group compared with the control group. Participants in the experimental group showed significant alleviation in psychological distress following the intervention, which was not observed in the control group. These findings are consistent with previous research demonstrating that slow breathing practices may reduce stress and anxiety through modulation of autonomic nervous system activity (1, 5, 6, 18). In addition, participants who practiced controlled deep breathing demonstrated improvements in executive functioning, as reflected in faster completion times on the TMT-B, a well-established neuropsychological measure of cognitive flexibility and visual–spatial processing efficiency (19). These findings suggest that breathing-based interventions may influence higher-order cognitive processes through mechanisms potentially related to arousal regulation and changes in resting-state neural activity.

Taken together, the results putatively indicate that controlled breathing techniques are associated with changes in resting-state neural oscillatory activity, executive functioning performance, and psychological distress measures in healthy young adults. The observed parallel changes in alpha/beta power ratio and psychological distress following the intervention suggest a potential relationship between neural and affective changes (20, 21). These findings provide preliminary support for the potential role of controlled breathing interventions in modulating brain activity and subjective psychological states in humans.

### 4.1 Limitations

Several limitations should be considered when interpreting the present findings. Primarily, although the sample size was sufficient to detect significant effects on resting EEG parameters, the relatively small number of participants per group may limit the generalizability of the results. Future studies with larger and more diverse samples (e.g., different age groups), including subclinical populations at increased risk for anxiety- and depression-related disorders, are warranted to further support the current findings (19).

In addition, the study examined the short-term effects of a single controlled-breathing session. It remains unclear whether repeated practice of controlled breathing techniques such as CDBT would produce longer-lasting neural-activity and psychological changes over extended periods following single- or multi-session interventions. Furthermore, although resting-state qEEG provides valuable insights into changes in neural oscillatory activity over specific periods or events, the current study did not include additional objective physiological stress markers, such as heart rate variability (HRV), breathing rate, cortisol levels, or electrodermal activity (EDA). Including such measures in future studies could further clarify the physiological mechanisms underlying the effects of CDBT on autonomic regulation and objective measures of neurophysiological distress (22). Furthermore, it is critical to examine in future studies changes in induced alpha power and across different frequency bands during closed eyes states (from closed-eyes onset over a five second interval) at parietal electrode sites to verify the effect is specific to ongoing alpha activity during close eye periods. Hence, to further support our findings, future research should also employ induced spectral power analysis during specific time-widows to verify that changes following CDBT are unique to ongoing resting alpha power, versus induced alpha power, or versus induced power changes in other frequency bands. Additionally, since CBDT was the main manipulation directly affecting respiratory patterns and related physiological measures (e.g., respiratory rate), future studies are encouraged to employ a respiratory belt allowing precise characterization of respiratory timing and amplitude across all participants. In relevance, it is important to note that the current study investigated the impact of an *instructed controlled-breathing procedure* on resting state oscillations rather than quantitatively establishing a relationship between changes in respiratory parameters and changes in resting neural oscillatory activity. Critically, respiratory, cardiovascular/autonomic, and ideally end-tidal CO₂ measurements should be employed in future studies focusing on identifying the physiological mechanisms underlying CDBT effects on resting EEG oscillatory activity, such as significant changes in mean parietal alpha power from pre to post intervention in the experimental group (20, 21, 23, 24).

Finally, and most importantly, the control condition consisted of natural breathing without an *active-control* intervention component to control for attention across all participants in the control condition. Therefore, future studies incorporating active control conditions matched for attentional engagement, expectancy, and verbal stimuli (presented vocally) are needed to determine whether the observed effects are specific to controlled breathing rather than effects of focused attention or lack of task engagement in the control group (19). In addition, our resting EEG assessments were restricted to electrodes of interest at specific frontal and parietal calp sites. A scalp-wide topographical analysis (e.g., 32 to 128 electrodes) would also be preferable to replicate the locality of observed effects at electrodes of interest examined in the current study.

## 5 Conclusion

The present study provides preliminary empirical support showing that a single-session CDBT intervention can modulate resting-state neural activity and reduce subjective psychological distress in healthy adults. Specifically, the breathing intervention was associated with increased alpha/beta power ratios at parietal sites and improvements in executive functioning. These findings add to the growing body of research on the psychophysiological effects of breathing-based interventions on human affective states and regulation of behavior (1, 22).

As hypothesized, the observed changes in neural oscillatory activity and affective-state perception were consistent with changes in emotional regulation following the intervention, suggesting that controlled deep breathing may influence both neurophysiological and affective-state measures of behavior. Controlled breathing techniques such as CDBT may therefore represent a simple behavioral strategy that supports reductions in psychological distress and cognitive control processes in adult humans. CDBT-effects on resting EEG parameters may reflect alterations in neural processes associated with emotional regulation and cognitive control (25). However, the specific neural pathways underlying these effects remain unclear and require further investigation using complementary neuroimaging (e.g., fMRI) and ANS measures (e.g., HRV). Furthermore, to verify the selective effects of CDBT on periodic components of alpha activity during closed eyes conditions, future investigations should note changes in the aperiodic component of the EEG spectrum that might influence observed effects on mean alpha power at parietal electrodes during closed eyes conditions. A more contemporary spectral analysis separating periodic oscillatory components from the aperiodic 1/f component would strengthen the neurophysiological interpretation of observed changes in parietal alpha power during closed eyes conditions (24).

In sum, given the use of a healthy sample, a single-session design, and the absence of additional physiological stress markers, further research is needed to examine whether CDBT produces similar effects in larger samples and in populations with elevated stress or psychological distress. Future studies should further investigate the long-term effects of CDBT and the neural mechanisms underlying changes in affective state perception and cognitive functioning, as well as its potential applicability in preventive interventions for individuals at increased risk for developing depression, anxiety, and stress-related symptoms (1, 6, 13).

## Data Availability

The raw data supporting the conclusions of this article will be made available by the authors, upon reasonable request.
